# CenSoc: Public Linked Administrative Mortality Records for Individual-level Research

**DOI:** 10.1038/s41597-023-02713-y

**Published:** 2023-11-15

**Authors:** Casey F. Breen, Maria Osborne, Joshua R. Goldstein

**Affiliations:** 1 University of California, Berkeley, Department of Demography, Berkeley, 94720 USA; 2 University of Oxford, Leverhulme Centre for Demographic Science and Department of Sociology, Oxford, OX1 UK

**Keywords:** Economics, Sociology

## Abstract

In the United States, much has been learned about the determinants of longevity from survey data and aggregated tabulations. However, the lack of large-scale, individual-level administrative mortality records has proven to be a barrier to further progress. We introduce the CenSoc datasets, which link the complete-count 1940 U.S. Census to Social Security mortality records. These datasets—CenSoc-DMF (N = 4.7 million) and CenSoc-Numident (N = 7.0 million)—primarily cover deaths among individuals aged 65 and older. The size and richness of CenSoc allows investigators to make new discoveries into geographic, racial, and class-based disparities in old-age mortality in the United States. This article gives an overview of the technical steps taken to construct these datasets, validates them using external aggregate mortality data, and discusses best practices for working with these datasets. The CenSoc datasets are publicly available, enabling new avenues of research into the determinants of mortality disparities in the United States.

## Background & Summary

The CenSoc datasets—so termed because they link the full-count 1940 Census (“Cen”) with Social Security Administration mortality records (“Soc”)—represent the first large-scale, nationally representative, publicly available data resource for researchers studying mortality. We constructed two datasets, the CenSoc-DMF (N = 4.7 million) and CenSoc-Numident (N = 7.0 million), both primarily composed of deaths over the age of 65. The scale and detail of CenSoc data allow researchers to make new discoveries in areas such as (i) mortality disparities by education, national origin, and race; (ii) early life conditions and later-life mortality; (iii) geographic variation and the neighborhood determinants of mortality; and (iv) natural experiments from local policies and chance events such as natural disasters. These research areas are of growing importance in understanding increases in disparities in life expectancy in the United States. Here, we describe how the CenSoc datasets were constructed, validate these datasets by benchmarking them against gold-standard aggregated mortality statistics, and discuss best practices for working with these datasets.

We are far from a complete understanding of the social determinants of longevity. Despite the longstanding interest in racial and class-based inequalities in health and mortality in the United States^1,2^, research is often limited by the lack of individual-level data^3,4^. Most research into the general dimensions of mortality disparities using individual-level data have relied on survey data, with sample sizes that preclude the analysis of smaller population subgroups such as the “oldest old” or minority populations. In the absence of comprehensive population-level registry data, researchers are increasingly turning to linked administrative datasets to answer some of the most pressing questions in social science research^3,5–8^. Yet many of these new linked administrative datasets—especially for mortality research—are only available in restricted contexts, limiting opportunities for replicating and extending analyses conducted with the data.

Fortunately, the data landscape for mortality researchers is improving. Recently, the U.S. Census Bureau has made available an internal, restricted-access version of the Numident, which has been linked to a series of economic, survey, and administrative data including the 1940, 2000, and 2010 Censuses^9^. These restricted data can be accessed in Federal Statistical Research Data Centers (FSRDC) and provide nearly complete mortality coverage from 1975 to the present. Another major data infrastructure project is the publicly available LIFE-M project, which links intergenerational census records to mortality records^10^. The LIFE-M linkages are based on a random sample of birth certificates in Ohio and North Carolina and track four generations of individuals longitudinally over the life course. These projects all represent important advances in data infrastructure for mortality researchers.

The comparative advantages of CenSoc are twofold. First, the CenSoc datasets are publicly available for unrestricted download, without the need for prior approval. This ensures that investigations with CenSoc data are reproducible and extendable. Second, the massive, nationally-representative sample allows researchers to conduct high-resolution mortality research, investigating disparities for smaller population subgroups and fine geographic areas. Early versions of CenSoc datasets have been used to make new findings concerning the long-run longevity benefits of education^11,12^, the impact of environmental disasters on mortality^13,14^, social insurance programs’ influence on later-life mortality^15^, and the relationship between homeownership and longevity^16^. These projects represent early applications of CenSoc data, and many opportunities remain.

## Methods

To construct the CenSoc datasets, we link the 1940 Census to two distinct sources of mortality data: the public Social Security Numident File (“Numident”) and the Social Security Death Master File (“DMF”). As there is no shared unique identifier between the 1940 Census and mortality records (e.g., a Social Security number), we use nominal record linkage algorithms to link the census and mortality records at the individual level^17,18^. We create two separate linked datasets—the CenSoc-DMF and the CenSoc-Numident—because the DMF and Numident have different fields available for record linkage and different mortality coverage windows. We describe the input datasets and our record linkage strategy below.

### Input datasets

#### 1940 census

The 1940 Census, conducted on April 1st, 1940, collected information on over 132 million Americans living in 44 million households. The 1940 Census form included 34 population questions and 31 housing questions and was the first U.S. census to include questions on wage and salary income, educational attainment, and detailed employment status. In addition, it collected information on exact street address, place of birth, citizenship, homeowner status, occupation, and more. The 1940 Census was also the first U.S. Census to leverage modern sampling techniques: every 20 person was asked 16 additional questions on topics such as their mother’s and father’s birthplace, veteran status, holder of a Social Security number, number of times married, age at first marriage, and number of children ever born. The 1940 Census was taken after the worst of the Great Depression and before the large war-time mobilization that soon followed, in a “business as usual” setting.

The 1940 Census records were made publicly available following the 72-year waiting period mandated by law. Following their public release, the Census records were digitized by the American genealogy company Ancestry and made available to the research community by IPUMS-USA^8,19^. Per an agreement with Ancestry, the public version of the 1940 Census from IPUMS-USA omits names and street addresses. A restricted version of the 1940 Census is available for researchers to access in restricted data enclaves, which includes names and street addresses. We use this restricted version of the 1940 Census to construct the CenSoc datasets, which we then publish using the public individual identifiers in the IPUMS 1940 Census public file.

#### Social security death master file

Our first set of death records comes from the Social Security Death Master File (DMF). The DMF has been used for both academic research^20^ and identity fraud prevention by financial services companies and government agencies. The DMF contains over 83 million death records, with nearly-complete coverage (95%+) from 1975–2005. Outside of this window, death coverage drops dramatically^21,22^. Each DMF death record contains full first and last name, exact date of birth (d/m/y), and exact date of death (d/m/y). The DMF does not contain information on gender or place of birth.

#### NARA social security numident file

Our second set of mortality records comes from the Social Security Numident File. The Numident is the backbone of the Social Security Administration’s record keeping system. For each person with a Social Security Number, the Numident tracks date of birth, date of death (if applicable), claims status, and other background information such as birthplace, race, sex, and parents’ first and last names. A subset of the Numident records was transferred to the National Archives and Record Administration (NARA) for public release. The public Numident contains nearly complete death coverage for Social Security Number holders between 1988 and 2005 and includes two additional record linkage fields not available in the DMF: place of birth and information on parents’ last names. Parents’ last names are especially valuable for record linkage because they enable researchers to determine a woman’s maiden name.

We cleaned and harmonized the public Numident application, claims, and death records into a single harmonized file: the Berkeley Unified Numident Mortality Database (BUNMD)^23^. This publicly-available file includes nearly 50 million death records but lacks the covariates available in the 1940 Census.

### Record linkage

To establish matches between the 1940 Census and mortality records, we use a deterministic record linkage algorithm, the “conservative” version of the ABE exact record linkage algorithm^24–26^. This linking strategy requires an exact match on standardized first name, last name, and place of birth (for Numident only). Priority is given to exact matches on age in 1940, with additional flexibility of up to ±2 years allowed. We use this conservative approach to establishing a match to prioritize minimizing the number of false matches over maximizing the total match rate^17^. As new matching methods emerge, we can create and release updated versions of these linkages, minimizing linkage errors with potential implications for the inferences drawn from these data^18^.

### Matching methods for women

Nominal record linkage is difficult for women because of changes from maiden to married names. In our setting, if a woman marries and changes her last name after the 1940 Census, her census and death records will have different last names, making it impossible to successfully establish a match. While we are not able to link women between the 1940 Census and the DMF, we are able to link women between the 1940 Census and the Numident using information on the father’s last name available in the Numident record. Specifically, we first identify marital status using information in the 1940 Census. For ever-married women, we link using last name in both the 1940 Census and the Numident, exactly the same as we do for men. For never-married women, we use their father’s last name in the Numident as a proxy for the last name they reported in the 1940 Census. (We note that this method cannot match women who re-married and changed their name again after 1940.)

### Statistical weights

To account for differences in inclusion probabilities by period, age, and demographic characteristics, we generate post-stratification weights using population totals from the Multiple Cause-of-Death (MCOD) mortality data from the National Vital Statistics System of the National Center for Health Statistics (NCHS)^27^. The MCOD datasets compile individual-level data from death certificates for all deaths that occur within the United States. The primary purpose of weights is to adjust for slightly worse coverage of younger ages of death within birth cohorts.

For people who were born in the contiguous United States (including the District of Columbia), died aged 65–100, and died during the years of 1988–2005 (CenSoc-Numident) or 1979–2005 (CenSoc-DMF), we weight up directly to population totals from NCHS data. For each dataset, individuals are split into cells cross-classified by year of death (*y*), age at death (*a*), sex (*s*), race (*r*), and birth state (*b*). We assign each person in a given cell a weight equal to the ratio of deaths in the NCHS data to deaths in the CenSoc data:

1 $${W}_{yasrb}=\frac{{\rm{number}}\;{\rm{of}}\;{\rm{deaths}}\;{\rm{in}}\;{\rm{NCHS}}\;{\rm{cell}}\;yasrb}{{\rm{number}}\;{\rm{of}}\;{\rm{deaths}}\;{\rm{in}}\;{\rm{CenSoc}}\;{\rm{cell}}\;yasrb}$$

To construct the weights, we use three race categories: Black, White, and Other. We are not able to use more detailed race categories due to comparability issues in race categories between the 1940 Census and the NCHS death certificates. We do not weight on Hispanic origin or ethnicity, as it is not directly available in the 1940 Census and was reported inconsistently across time and place in the NCHS death certificates.

The universe of deaths for CenSoc and NCHS data differ slightly. CenSoc data captures individuals with Social Security numbers (SSNs), including people with SSNs dying abroad. In contrast, NCHS data may include non-residents and non-SSN holders that died in the U.S. The presence of non-SSN holders in NCHS data, in addition to immigrants who entered the country after the 1940 census, are mainly problematic for weighting decedents born abroad. We assign immigrants alternative weights. We are not able to assign weights to those born in America and dying abroad because we cannot identify deaths that occur abroad. However, such deaths are small in number, ranging from several thousand to tens of thousands^9,28^. While the age and birthplace compositions of persons dying abroad are largely unknown, the absence of such deaths in NCHS data may slightly deflate weights among age 65+ American-born SSN holders.

#### Non-standard weights

A portion of CenSoc data cannot be directly weighted using the weighting strategy described above. We address the following types of problematic records as follows:

- Deaths in the years 1975–1978: Birth state of decedents is not available in MCOD data from 1975–1978. For years prior to 1979, we assign the same weight for age/race/birthplace strata as in 1979.
- Decedents born abroad, in current U.S. territories, Alaska, and Hawaii: Any person born outside the 48 contiguous United States and the District of Columbia is only observable in CenSoc if they moved to the contiguous U.S. before census day in 1940. NCHS totals for these groups are inappropriate to use for weighting, as immigrants who entered the country after 1940 are included, as well as all Alaskans and Hawaiians. Instead, we assign records the mean weight of U.S.-born decedents of the same year, age, race, and sex.
- Records not weightable due to other data issues: A very small number of records cannot be weighted directly due to issues like missing birthplace or because they belong to a stratum not present in NCHS population data. We first attempt to assign these records the mean weight of U.S-born decedents of the same age, year, sex, and race. If this fails, records are given a weight of 1 (necessary for less than 600 records in each data set).

#### Weighting adjustments

The unadjusted weighted CenSoc deaths to American-born decedents from 1979–2005 total only 99.5% of NCHS death tallies, due to presence of age/year/sex/race/birthplace cells extant in NCHS data but not captured by CenSoc. To address bias introduced by empty cells, we utilize raking ratio estimation^29^, as implemented with the R package Survey^30^, to calibrate weighted marginal totals to population marginal totals by year, age, race, sex, and birthplace.

The raw post-stratification weights also contain extreme weights. We trim weights to a minimum of 1 and a maximum of 5 times the mean unadjusted weights to reduce the potential impact of extremely high weights on analyses. Less than 1% of raw weights fell above the maximum threshold. Weights for the years 1975–1978 and the foreign-born are trimmed but not otherwise calibrated or adjusted due to absence of useable population totals. For American-born decedents in the years 1979–2005, records are iteratively raked and trimmed until weights are both calibrated to population marginal totals and fall within an acceptable range.

## Data Records

### CenSoc data download

The CenSoc datasets and their accompanying documentation are publicly available for download from the Harvard Dataverse^31,32^. Researchers must sign an agreement to properly cite and not redistribute the data. Table 1 provides an overview of the key features of the CenSoc-DMF and CenSoc-Numident datasets.

**Table 1:** Characteristics of CenSoc Datasets.

	CenSoc-DMF	CenSoc-Numident
Gender	Men-Only	Men and Women
1940 Census Covariates	Yes	Yes
High Coverage of Deaths	1975–2005	1988–2005
Size	4.7 Million	7.0 Million

#### Censoc-DMF

The CenSoc-DMF file is comprised of approximately 4.7 million person-level records and 7 variables^31^. The *histid* variable uniquely identifies each record in the dataset. The other variables report statistical weights, dates of birth, and dates of death (see Table 2). The CenSoc-DMF file only includes men, as surname changes at the time of marriage preclude accurate linkage of women. To access the 50+ variables available in the 1940 Census, such as census race, education, wage and salary income, small area geographic identifiers, and occupation, researchers must link the CenSoc-DMF onto the publicly-available IPUMS 1940 Census on the unique identifier *histid*.

**Table 2:** Variables in the CenSoc-DMF file.

Variable	Description
histid	Unique historical identifier
byear	Year of birth
bmonth	Month of birth
dyear	Year of death
dmonth	Month of death
death_age	Age of death (years)
weight	Person-level weight

Linkage to the 1940 Census adds all individual-level Census variables, including education, wage and salary income, and residential context.

#### CenSoc-numident

The CenSoc-Numident file is comprised of approximately 7.0 million records and 18 variables^32^. The *histid* variable is a unique identifier that is also available in the public 1940 Census records. The other variables report a statistical weight, date of birth, date of death, birthplace, race, and ZIP code of residence at time of death (see Table 3). The CenSoc-Numident file contains both men and women. To access the 50+ variables available in the 1940 Census (e.g., census race, education, wage income, small area identifiers, and occupation), investigators must link the CenSoc-Numident onto the publicly-available IPUMS 1940 Census on the unique identifier *histid*.

**Table 3:** The variables in the CenSoc-Numident file.

Variable	Description
histid	Historical unique identifier
byear	Year of birth
bmonth	Month of birth
dyear	Year of death
dmonth	Month of death
death_age	Age at death (years)
sex	Sex
race_first	Race on first application
race_first_cyear	First race: application year
race_first_cmonth	First race: application month
race_last	Race on last application
race_last_cyear	Last race: application year
race_last_cmonth	Last race: application month
bpl	Place of birth
zip_residence	ZIP code of residence at time of death
socstate	State where Social Security Number was issued
age_first_application	Age at first Social Security application
weight	Person-level weight

Linkage to the 1940 Census adds all individual-level Census variables, such as educational attainment, wage and salary income, and residential context.

## Technical Validation

### Validation of mortality rates

As a validation exercise, we estimate the cohort age-specific mortality rates from the CenSoc datasets and benchmark them against the Human Mortality Database^33^, the world’s leading scientific data resource on aggregate mortality statistics in developed countries. To estimate the age-specific cohort mortality rate, we use the extinct cohort method^34–36^. Specifically, within a given cohort, we calculate the total number of survivors at a given age by summing up all the deaths which occurred above that age. For cohorts that are not extinct by the end of our mortality observation window, such as the cohort of 1910, we estimate the additional number of cohort deaths occurring after our mortality observation window ends using the Human Mortality Database cohort exposure-to-risk data^33^. We then calculate the age-specific mortality rates from age-specific ratios of deaths to survivors.

**Fig. 1:**
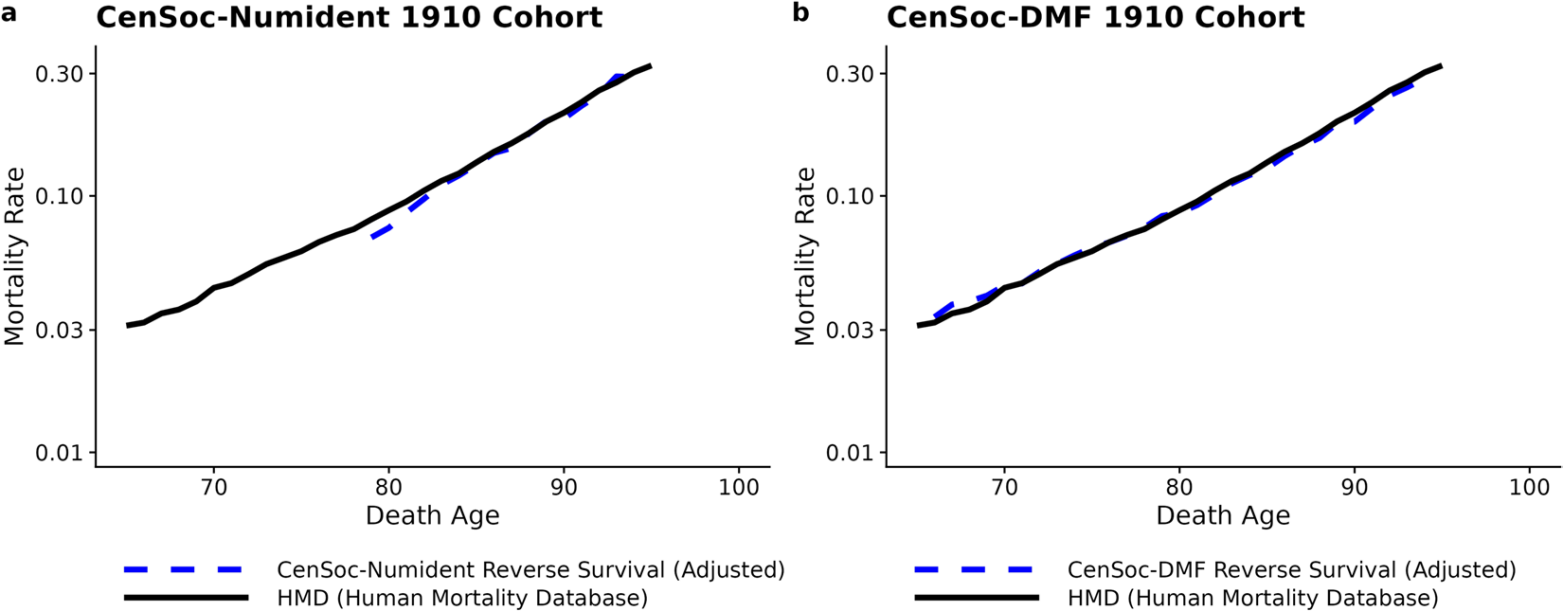
Estimated age-specific mortality rates for the cohort of 1910 from the CenSoc-Numident (panel **a**) and CenSoc-DMF (panel **b**) benchmarked against the cohort age-specific mortality rates from the Human Mortality Database (HMD). The cohort age-specific mortality rates from the CenSoc datasets were calculated using the extinct cohort method. **Note**: Figures are given in the log-scale.

Figure 1 shows the estimated age-specific mortality rate benchmarked against the age-specific mortality rate from the Human Mortality Database (HMD) for the cohort of 1910. The estimated age-specific mortality rates align closely for both the CenSoc-DMF and the CenSoc-Numident, demonstrating the aggregate mortality rates from the CenSoc datasets closely replicate gold-standard age-specific mortality estimates. We would not expect identical mortality rates from the two data sources as the CenSoc matches do not include post-1940 immigrants, while the HMD is influenced by migration effects after 1940.

### Validation of mortality estimates

The CenSoc datasets allow researchers to investigate the relationship between early life sociodemographic characteristics and later-life longevity^11,13,16,37^. To validate estimates of the association between covariates and longevity using CenSoc data, we present an updated example from Goldstein *et al*.^38^ on the association between education and longevity. Table 4 compares the estimated educational gradient in longevity from the CenSoc datasets to external estimates from three other studies relying on different sources of mortality data^12,39,40^. The estimated education gradient in longevity from CenSoc-DMF and CenSoc-Numident align closely with the external educational gradient estimates from the other three other studies. This agreement demonstrates that the CenSoc datasets can produce reliable estimates of the association between covariates and longevity.

**Table 4:** Estimates of the association between an additional year of schooling and mortality for men from four studies.

Study	Dataset	Birth Cohorts	Death Window	Method	Hazard Ratio	e(35)
Lleras-Muney *et al*.^12^	Census Tree	1906–1915	1941–2019	OLS	—	0.404 (0.355, 0.452
Halpern-Manners *et al*.^39^	SSDMF-NUMIDENT linked to 1940 Census	1910–1920	1960–2013	OLS	—	0.387 (0.379, 0.395)
Rogers *et al*.^40^*	National Health Interview Survey (NHIS) Linked Mortality Files	1908–1928	1997–2002	Cox PH Model	0.945	0.544
This Study (2022)	CenSoc-DMF	1910–1920	1975–2005	Gompertztrunc	0.961 (0.961, 0.962)	0.473 (0.464, 0.482)
This Study (2022)	CenSoc-Numident	1910–1920	1988–2005	Gompertztrunc	0.958 (0.956, 0.959)	0.521 (0.507, 0.535)

Despite substantial differences in study design-each study uses different birth cohorts, methods, and mortality data—estimates generally align closely across studies.

The “OLS” method corresponds to the Ordinary Least Squares (OLS) regression on age of death,“Cox PH Model” corresponds to the Cox proportional hazards model, and “Gompertztrunc” corresponds to the parametric Gompertz method introduced in [38]. We convert hazard ratios to estimates of *e*(35), assuming deaths follow a Gompertz distribution with parameters *a* = 3.34 × 10^−5^ and *b* = 0.1.

*We calculate the effect of an additional year of education from the Rogers *et al*.^40^ study by assuming those in the “less than 12 years of education” category had on average 8 years of education. We assume those in the “BA” category had 16 years of education, 8 years more than those in the “less than 12 year of education” category. We then calculate the annualized hazard rate as: $h{r}_{(annualized)}={\left(h{r}_{({\rm{ba}})}/h{r}_{( < {\rm{12yrs}})}\right)}^{1/8}={(0.748/1.178)}^{(1/8)}=0.9448$. The Halpern-Manners *et al*.^39^ estimate includes controls for occupation category, family size, U.S. born parents, dummies for age at 1940 census, and race. The Lleras-Muney *et al*.^12^ estimates are from a regression including birth year and state-of birth fixed effects.

### Validation of mortality coverage

**Fig. 2:**
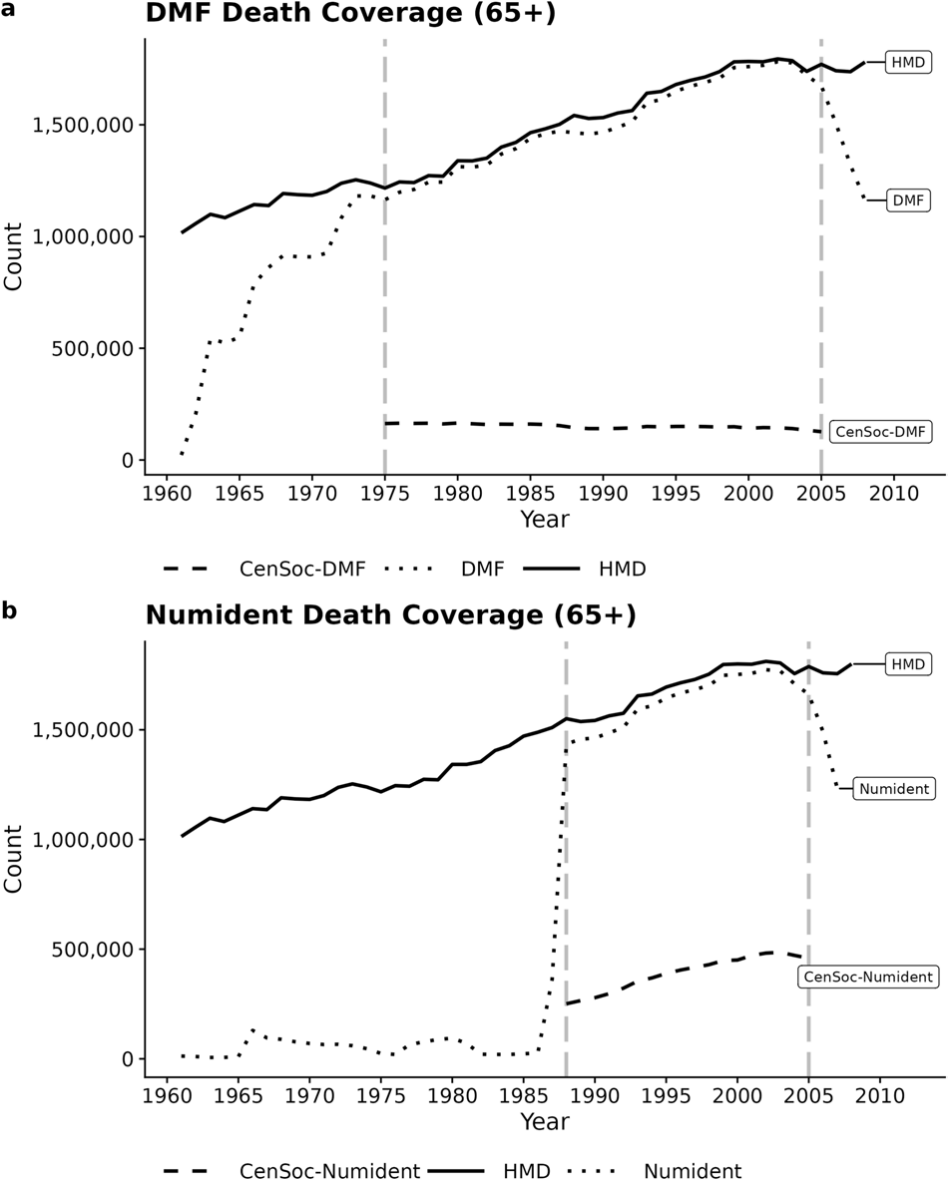
Mortality coverage of the unweighted Numident (panel **a**) and unweighted DMF (panel **b**) datasets. The gray vertical dashed lines bound the high mortality coverage window for each respective dataset. The weighted CenSoc-DMF and CenSoc-DMF counts (not shown) are generally slightly lower than total population (HMD) deaths, as the HMD figures include post-1940 immigration.

As a validation exercise, we benchmark the number of deaths captured in the original Numident and DMF mortality files against the Human Mortality Database (HMD) totals^33^. The mortality coverage for deaths to individuals 65+ is over 95% in the Numident (Fig. 2a) between 1988–2005 and over 95% in the DMF (Fig. 2b) between 1975–2005. Outside these windows, mortality coverage is very low. We restrict our universe of potential matches for the CenSoc-DMF and CenSoc-Numident datasets to deaths occurring in the high mortality coverage window.

We match 22% of Numident records and 17% of DMF records to the 1940 Census. This match rate is comparable to other efforts linking historical data^26^. We note that our primary focus is ensuring the accuracy and representativeness of the matches rather than maximizing the overall number of matches. Weights can account for differential mortality coverage over time.

**Fig. 3:**
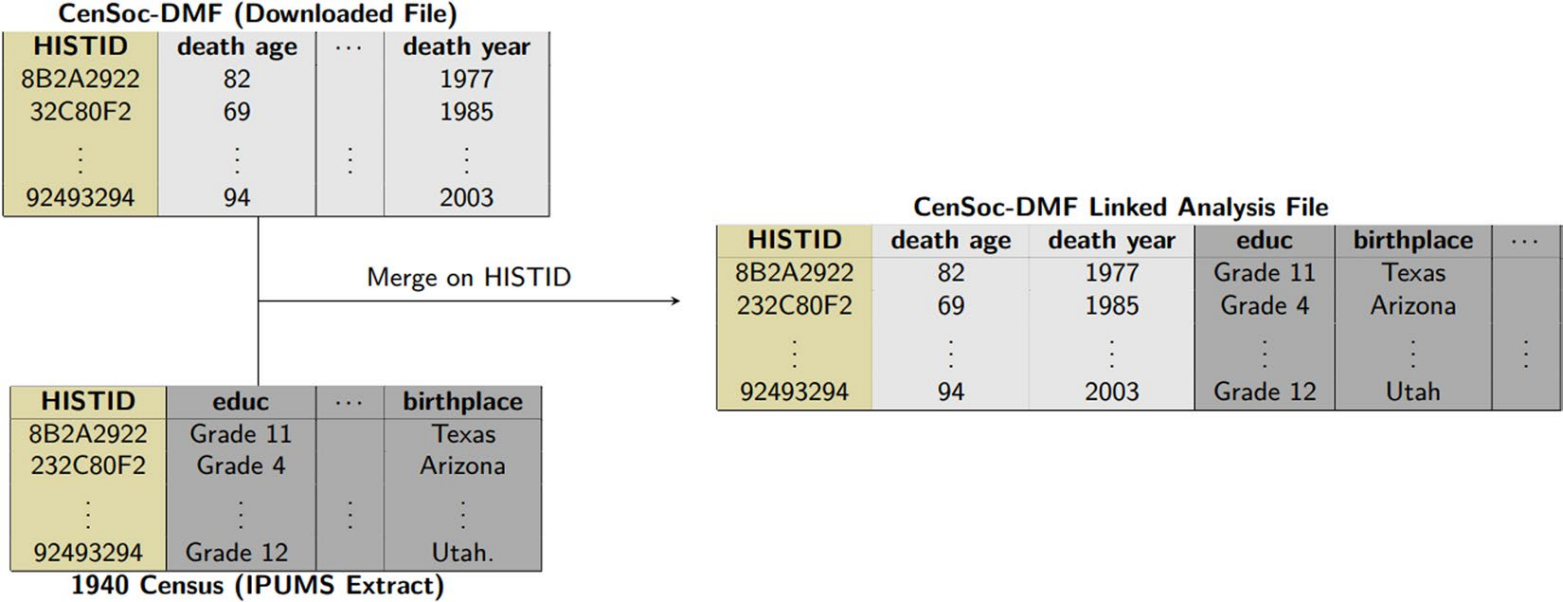
Illustration of merging the CenSoc-DMF to an IPUMS 1940 Census data extract on HISTID, a shared unique identifier in both files. Columns shaded light grey represent information only in the CenSoc-DMF file; columns shaded dark grey represent information only available in the 1940 Census.

### Validation of matching

#### Representativeness of matches

The representativeness of our CenSoc matches can be assessed relative to those who died in the coverage period and/or those alive in 1940. Our weighting strategy assures representativeness with respect to deaths. Even with a representative set of deaths, our nominal record linkage procedure will introduce some selection into our final matches. We can compare the characteristics of individuals enumerated in the 1940 Census to the subset of individuals successfully matched in the CenSoc datasets. The representativeness of the CenSoc matches for the pooled birth cohorts of 1900–1920 is presented in Tables 5–7. The CenSoc-DMF and CenSoc-Numident datasets reflect the general population but slightly overrepresent higher socioeconomic status individuals. For instance, the percentage of men in the CenSoc-DMF who did not complete high school (62.5%) is slightly lower than the general population (65.3%). Black people are underrepresented in both datasets, comprising 9.6% of the general male population but only 3.9% of the CenSoc-DMF and 5.0% of men in the CenSoc-Numident. Weights help correct for some of this underrepresentation: 8.9% of the men in weighted CenSoc-DMF and 7.8% of the men in the weighted CenSoc-Numident are Black, aligning more closely to the 9.6% of men who are Black in the 1940 Census. Additionally, despite the lower match rate, the sociodemographic characteristics of matched Black individuals closely align with those of the general Black population^23^.

**Table 5:** Representativeness of men in the CenSoc-DMF for pooled birth cohorts of 1900–1920.

	Count		Proportion (%)			Difference (%)
	CenSoc-DMF	1940 Census (men)	CenSoc-DMF (unweighted)	CenSoc-DMF (weighted)	1940 Census (men)	Weighted DMF minus Census
**Education**						
<High School	1,579,575	14,408,138	62.5	64.4	65.3	−0.9
High School or some college	732,893	5, 836, 119	29.0	27.3	26.5	0.8
Bachelor’s Degree	113,433	896, 945	4.5	4.3	4.1	0.2
Advanced Degree	55,402	410,006	2.2	2.1	1.9	0.2
NA	44, 199	508, 970	1.8	1.9	2.3	−0.4
**Race**						
White	2,415,637	19,828,647	95.6	90.7	89.9	0.8
Black	97, 889	2,114,858	3.9	8.9	9.6	−0.7
Other	11,976	116,673	0.5	0.4	0.5	−0.1
**Marital Status**						
Married	1,582,302	13,629,223	62.7	64.4	61.8	2.6
Not married	943, 200	8, 430, 955	37.3	35.6	38.2	−2.6
**Home Ownership**						
Home Owner	1,002,411	7, 966, 734	39.7	38.2	36.1	2.1
Not Home Owner	1,523,091	14,093,444	60.3	61.8	63.9	−2.1
**Socioeconomic Index**						
1–9	426, 344	4, 256, 451	16.9	18.2	19.3	−1.1
10–14	330, 941	2, 802, 564	13.1	14.0	12.7	1.3
15–25	679, 188	5, 626, 587	26.9	26.4	25.5	0.9
26+	904, 882	7, 376, 232	35.8	34.2	33.4	0.8
NA	184,147	1,998,344	7.3	7.1	9.1	−2.0
**Rural**						
Rural	1,065,217	9,315,391	42.2	43.8	42.2	1.6
Urban	1,460,285	12,744,787	57.8	56.2	57.8	−1.6
**Region**						
East North Central	626, 058	4, 458, 018	24.8	21.0	20.2	0.8
East South Central	127,530	1,730,137	5.0	7.6	7.8	−0.2
Middle Atlantic	563, 691	4, 729, 396	22.3	21.1	21.4	−0.3
Mountain	81,371	695, 813	3.2	3.1	3.2	−0.1
New England	162,172	1,349,277	6.4	6.2	6.1	0.1
Pacific	220, 194	1,747,215	8.7	8.0	7.9	0.1
South Atlantic	226, 257	3, 022, 156	9.0	12.7	13.7	−1.0
West North Central	313, 891	2,136,322	12.4	10.4	9.7	0.7
West South Central	204, 338	2,191,844	8.1	9.9	9.9	0.0

Columns compare the number and proportion of people with certain sociodemographic characteristics in the CenSoc-DMF to the male 1940 Census population. The rightmost column is the proportion of people in the weighted CenSoc data with a characteristic minus the proportion of people in the 1940 census with that characteristic. A difference of 0 means that the CenSoc-DMF contains the same proportion of people with that characteristic as the 1940 Census. A negative difference indicates that a group is underrepresented in the CenSoc-DMF compared to the 1940 Census, and a positive difference indicates a group is overrepresented in the CenSoc-DMF.

**Note**: The 1940 Census does not include information about year of birth, so we approximate birth cohort from reported age in the 1940 Census.

**Table 6:** Representativeness of men in the CenSoc-Numident for pooled birth cohorts of 1900–1920.

	Count		Proportion (%)			Difference (%)
	CenSoc-Numident	1940 Census	CenSoc-Numident (unweighted)	CenSoc-Numident (weighted)	1940 Census	Weighted Numident minus Census
**Education**						
<High School	674,661	14,408,138	52.5	57.8	65.3	−7.5
High School or some college	506,686	5, 836, 119	39.4	32.9	26.5	6.4
Bachelor’s Degree	56,782	896, 945	4.4	5.0	4.1	0.9
Advanced Degree	24,282	410,006	1.9	2.4	1.9	0.5
NA	22, 164	508, 970	1.7	1.9	2.3	−0.4
**Race**						
White	1,215,794	19,828,647	94.6	91.8	89.9	1.9
Black	63,596	2,114,858	5.0	7.8	9.6	−1.8
Other	5, 185	116,673	0.4	0.4	0.5	−0.1
**Marital Status**						
Married	522, 565	13,629,223	40.7	54.3	61.8	−7.5
Not Married	762, 010	8, 430, 955	59.3	45.7	38.2	7.5
**Home Ownership**						
Home Owner	534, 566	7, 966, 734	41.6	38.7	36.1	2.6
Not Home Owner	750, 009	14,093,444	58.4	61.3	63.9	−2.6
**Socioeconomic Index**						
1–9	246, 514	4, 256, 451	19.2	18.4	19.3	−0.9
10–14	131,375	2, 802, 564	10.2	11.7	12.7	−1.0
15–25	349, 093	5, 626, 587	27.2	26.0	25.5	0.5
26+	409, 063	7, 376, 232	31.8	34.6	33.4	1.2
NA	148,530	1,998,344	11.6	9.2	9.1	0.1
**Rural**						
Rural	556, 190	9,315,391	43.3	42.9	42.2	0.7
Urban	728, 385	12,744,787	56.7	57.1	57.8	−0.7
**Region**						
East North Central	291,491	4, 458, 018	22.7	20.9	20.2	0.7
East South Central	72, 772	1,730,137	5.7	7.2	7.8	−0.6
Middle Atlantic	256, 778	4, 729, 396	20.0	21.0	21.4	−0.4
Mountain	49, 650	695, 813	3.9	3.4	3.2	0.2
New England	99, 757	1,349,277	7.8	7.0	6.1	0.9
Pacific	115,768	1,747,215	9.0	8.5	7.9	0.6
South Atlantic	136,447	3, 022, 156	10.6	12.2	13.7	−1.5
West North Central	154,759	2,136,322	12.0	10.3	9.7	0.6
West South Central	107,153	2,191,844	8.3	9.6	9.9	−0.3

Columns compare the number and proportion of people with certain sociodemographic characteristics in the CenSoc-Numident to the male 1940 Census population. The rightmost column is the proportion of people in the weighted CenSoc data with a characteristic minus the proportion of people in the 1940 census with that characteristic. A difference of 0 means that the CenSoc-Numident contains the same proportion of people with that characteristic as the 1940 census. A negative difference indicates that a group is underrepresented in the CenSoc-Numident compared to the 1940 census, and a positive difference indicates a group is overrepresented in the CenSoc-Numident.

**Note**: The 1940 Census does not include information about year of birth, so we approximate birth cohort from reported age in the 1940 Census.

**Table 7:** Representativeness of women in the CenSoc-Numident for pooled birth cohorts of 1900–1920.

	Count		Proportion (%)			Difference (%)
	CenSoc-Numident	1940 Census	CenSoc-Numident (unweighted)	CenSoc-Numident (weighted)	1940 Census	Weighted Numident minus Census
**Education**						
<High School	973,154	13,843,779	53.7	58.2	61.0	−2.8
High School or some college	725,184	7, 376, 530	40.0	35.2	32.5	2.7
Bachelor’s Degree	70,641	819,838	3.9	4.0	3.6	0.4
Advanced Degree	13,340	175,240	0.7	0.8	0.8	0.0
NA	31,468	477, 719	1.7	1.8	2.1	−0.3
**Race**						
White	1,716,543	20,213,119	94.6	91.6	89.1	2.5
Black	92, 905	2, 407, 423	5.1	8.2	10.6	−2.4
Other	4, 339	72, 564	0.2	0.3	0.3	0.0
**Marital Status**						
Married	1,202,085	16,207,977	66.3	73.1	71.4	1.7
Not Married	611,702	6, 485, 129	33.7	26.9	28.6	−1.7
**Home Ownership**						
Home Owner	702, 573	8, 249, 277	38.7	38.9	36.4	2.5
Not Home Owner	1,111,214	14,443,829	61.3	61.1	63.6	−2.5
**Socioeconomic Index**						
1–9	67, 698	1,173,667	3.7	3.8	5.2	−1.4
10–14	17,506	332, 961	1.0	1.1	1.5	−0.4
15–25	188,757	2,441,903	10.4	8.9	10.8	−1.9
26+	348, 684	3, 909, 172	19.2	16.3	17.2	−0.9
NA	1,191,142	14,835,403	65.7	69.8	65.4	4.4
**Rural**						
Rural	739, 503	8, 794, 855	40.8	41.8	38.8	3.0
Urban	1,074,284	13,898,251	59.2	58.2	61.2	−3.0
**Region**						
East North Central	398, 724	4, 538, 495	22.0	20.4	20.0	0.4
East South Central	124,553	1,830,093	6.9	8.0	8.1	−0.1
Middle Atlantic	370, 900	4, 965, 835	20.4	20.7	21.9	−1.2
Mountain	59, 503	670, 518	3.3	3.0	3.0	0.0
New England	131,049	1,423,222	7.2	6.5	6.3	0.2
Pacific	136,263	1,659,178	7.5	7.1	7.3	−0.2
South Atlantic	217,393	3,136,993	12.0	13.4	13.8	−0.4
West North Central	207, 603	2,180,182	11.4	10.5	9.6	0.9
West South Central	167,799	2, 288, 590	9.3	10.3	10.1	0.2

Columns compare the number and proportion of people with certain sociodemographic characteristics in the CenSoc-Numident to the femlae 1940 Census population. The rightmost column is the proportion of people in the weighted CenSoc data with a characteristic minus the proportion of people in the 1940 Census with that characteristic. A difference of 0 means that the CenSoc-Numident contains the same proportion of people with that characteristic as the 1940 census. A negative difference indicates that a group is underrepresented in the CenSoc-Numident compared to the 1940 census, and a positive difference indicates a group is overrepresented in the CenSoc-Numident.

**Note**: The 1940 Census does not include information about year of birth, so we approximate birth cohort from reported age in the 1940 Census.

The representativeness of the matches has implications for inference. If the under or overrepresented population subgroups differ on the outcome of interest, this may lead to biased estimates of population-level parameters^18^. To address this, researchers can conduct stratified analyses (e.g., fit separate models for Black and White subgroups). However, the errors introduced by sample non-representativeness are generally modest compared to errors introduced by false matches^17,18^.

#### Validation using middle initials

To assess the accuracy of matches in the absence of ground-truth data, we investigate the agreement between the middle initials reported in the Census and the mortality record. As middle initial was not used as a matching field, we interpret the rate of disagreement on middle initials as an upper bound for the false match rate. Disagreements on middle initials may reflect an actual false match or a correct match where middle initials disagree due to reporting errors, transcription errors, or digitization errors. We use middle initials rather than full middle names because full middle names are rarely available in both Census and mortality records. We restrict our analysis to men to avoid complications with middle name changes at the time of marriage for women.

In the CenSoc-Numident, middle initials are available for 78% of Numident records, 30% of 1940 Census records, and 27% of records in both datasets. Of the 27% of records that have a middle initial in both datasets, middle initial agrees in 87% of the records. Middle initials are available for 43% of records in the DMF, 30% of records in the 1940 Census, and 15% of records in both datasets. Middle initials agree in 85% of matches in the CenSoc-DMF. Given the high rate of transcription errors in the 1940 Census^26^, the middle-initial agreement rate of 85% suggests an even higher level of correct matches.

## Usage Notes

### Linking censoc files with the ipums 1940 census

Researchers can download the public CenSoc files and link them with the complete-count 1940 Census on the unique identifier *histid*, which is available in both datasets (Fig. 3). Researchers can freely download a copy of the 1940 Census from IPUMS-USA^19^. Custom data extracts of the 1940 Census can be obtained by creating an account at https://usa.ipums.org/usa/https://usa.ipums.org/usa/ and selecting variables of interest (including *histid*) to download. Census data extracts may take several hours to be generated and take additional time to download. Researchers can alternatively access a restricted version of the complete-count 1940 Census at one of the IPUMS-approved secure data enclaves. The secure version of the 1940 Census includes first and last names and street-level addresses in addition to all the covariates in the public 1940 Census. In addition, researchers can download a prelinked “demo” versions of the CenSoc files, containing a 1% sample of the complete CenSoc datasets with 20 mortality covariates from the 1940 Census^41^.

### Mortality estimation

One technical limitation of the CenSoc datasets is that they include records for individuals who have died, without information on survivors. In addition, the datasets only include deaths for a left and right (“doubly”) truncated window. This situation of having “deaths without denominators” precludes the calculation of occurrence-exposure mortality rates and the use of the conventional tools of individual-level survival analysis^21^. In the presence of double-truncation, methods such as Cox-Proportional hazards methods or linear regression on age of death will result in attenuated estimates of regression coefficient^38^.

If researchers use conventional regression methods, they should keep in mind first that their coefficients will be biased toward zero (attenuated). Second, it is important that researchers include fixed effects for year of birth. We recommend fitting regressions of the form:

2 $${\rm{a}}{\rm{g}}{\rm{e}}\,{\rm{o}}{\rm{f}}\,{{\rm{d}}{\rm{e}}{\rm{a}}{\rm{t}}{\rm{h}}}_{i}={\beta }_{0}+{\gamma }_{t}{t}_{i}+\beta {Z}_{i}+{\epsilon }_{i},$$

where *β*_0_ is a general intercept, *γ*_*t*_ is the intercept for individuals born in year *t*, and **β** is the effect of a covariate *Z*_*i*_ on age of death. The birth year fixed effects are crucial to include because people born earlier will be observed dying at older ages.

We have also developed open-source software in the R language to estimate unbiased effects on mortality rates subject to double truncation of death counts^38^. The package **Gompertztrunc** is available to download at https://cran.r-project.org/web/packages/gompertztrunc/index.htmlCRAN. This approach assumes mortality follows a parametric Gompertz distribution and uses maximum likelihood estimation techniques to estimate mortality differentials. Specifically, we assume mortality follows a parametric Gompertz hazard model where the likelihood associated with a set of observed ages of death *x*_*i*_ with parameters *θ* (e.g., the intercept and slope of the log-Gompertz curve, which may themselves be functions of covariates) is given by the product of the normalized densities, with truncation on the right at age ${x}_{i}^{r}$ and on the left at age ${x}_{i}^{l}$:

3 $$L(\theta )=\prod _{i}{L}_{i}(\theta )=\prod _{i}\frac{f({x}_{i}| \theta )}{F({x}_{i}^{r}| \theta )-F({x}_{i}^{l}| \theta )},$$

where *f* is the density and *F* is the cumulative distribution.

For example, a proportional hazards model for the effect of covariates on mortality for individual *i* aged *x* with covariates *Z*_*i*_ assuming baseline Gompertz hazards is given by:

4 $${h}_{i}(x| \beta )={a}_{0}{e}^{{b}_{0}x}{e}^{\beta {Z}_{i}},$$

where *α*_0_ and *b*_0_ are baseline Gompertz parameters. In this case, the observed data would contain for each person values *x*_*i*_ for the age of death, *Z*_*i*_ for covariates (e.g., years of education, place of birth), and the right and left truncation ages ${x}_{i}^{r}$ and ${x}_{i}^{l}$ for each cohort. The model estimates would be the parameter values ${\widehat{a}}_{0},{\widehat{b}}_{0}$ and $\widehat{\beta }$. For a more comprehensive discussion of this method, please see Goldstein *et al*.^38^.

When possible, we recommend researchers work with this parametric Gompertz approach designed for estimating mortality disparities in the presence of double truncation. If researchers analyze CenSoc data using conventional methods such as OLS regression on age of death, they must clearly state that the estimated regression coefficients are attenuated by the double truncation.

### Research outside high coverage time periods

To date, most efforts involving weighting and mortality analysis have been developed for the high-coverage period over age 65. This roughly corresponds to birth cohorts of 1900–1925 for CenSoc-DMF and birth cohorts of 1910–1925 for CenSoc-Numident. Although it is possible to work with birth cohorts outside of this window, researchers should proceed with caution, spending extra time and effort on weighting and mortality estimation methods.

### Linkage to other datasets

The CenSoc datasets can also be linked onto other census or administrative records. For instance, researchers can take advantage of recent advances in census linkage infrastructure. Both the IPUMS Multi-Generational Longitudinal Panel Project (IPUMS-MLP)^19^ and the Census Linkage Project^42^ have publicly released crosswalks linking the complete-count decennial censuses from 1870–1940. Using these resources, researchers can track individuals in CenSoc longitudinally throughout their life course. In addition, researchers can also link CenSoc onto other datasets using the matching fields available in the CenSoc datasets. We have recently publicly released the CenSoc Army Enlistment Records, which link the CenSoc datasets to the World War II Army Records (N = 9 million) to obtain new covariates such as height, weight, and army rank. We also plan to link both the DMF and Numident mortality records onto the 1950 Census following its release from IPUMS.

## Data Availability

The original scripts to clean, process, and match the original 1940 Census and mortality records were written in the R programming language. They are available at https://github.com/caseybreen/censocdevgithub.com/caseybreen/censocdev. The code to reproduce all figures and tables in this paper is available from the Open Science Framework^43^.
